# A Plea for Civility

**DOI:** 10.1002/ece3.74395

**Published:** 2026-09-15

**Authors:** Allen J. Moore, Arley Muth

**Affiliations:** ^1^ Department of Entomology University of Georgia Athens Georgia USA; ^2^ John Wiley & Sons Hoboken New Jersey USA

The word “unprecedented” has become an overused term since 2020; however, it really does feel like we are living in unprecedented times. Science, particularly blue‐sky research, is under attack in countries that previously supported and celebrated the creation of new knowledge. Universities appear to be facing crises on a weekly basis. Academia, once the posterchild for stability, has become an uncertain workplace where the rules seemingly change randomly, there are words in grant applications that can get funding terminated, and the existence of entire fields of study is threatened. It is understandable there is heightened anxiety, fear, and anger amongst our colleagues. We understand, and we support all of you in these difficult times. That said, we are also appealing to you all to remember that everyone is living in these uncomfortable times, and words that are communicated—the reason we exist as a journal—can support or can destroy. Here, we ask that you think carefully about how you phrase your interpretation of previous work, peer‐review commentaries, and ideas in recognition that we wish to promote science constructively and equitably.

As reviewers, it is easy to write remarks that can be unintentionally misinterpreted. We certainly do not wish to censor or dictate how reviews are constructed but we do ask that you consider the receiver. Ad hominem comments and implying you understand intent is inappropriate and authors reserve their right to clarify and justify their intentions. Assume any errors that you find (and after all, that is really what we want from our reviewers) are unintentional, uninformed, or made through innocent misunderstanding, not intentional neglect. This is nearly always the case. Above all, do not assume that you know why an author has chosen to present their work in a particular way. It is rarely appropriate to suggest “a better presentation” unless you justify how this helps remove ambiguity or potential for misunderstanding. Remember that our authors (and audience) is international and often English is not their first language. Commenting on the standard of English is inappropriate; providing helpful edits is welcome. We remain committed to being an “author‐friendly journal”, eclectic and accommodating in style and presentation, and encourage you to look for reasons to publish, not reasons to reject a paper. We encourage submissions of sound science, interesting ideas, not only standardized reports. We rely on the expertise and the good will of our reviewers, whom we respect and applaud. We listen to our reviewers and use their reviews to improve papers that can be improved and, yes, reject those that need further incubation. At the same time, we ask that reviewers understand that authors have put time, effort, and often ego into their work. Reviews can hurt as much as help. Close and critical reviews are necessary for the peer‐review process, but we hope they will be developmental and not devastating.

For our authors, the term “author‐friendly” was never meant to imply authors were free to write unwarranted or harsh opinions within their manuscripts. If an author presents a viewpoint, especially a controversial or contrary one, it should be evidence‐based with the evidence clearly presented and the viewpoint clearly presented as opinion, not fact. If it is contrary to the opinion or presentation of others, it is not appropriate to be critical of those who hold opposing views. You can criticize the perspective but not the holder. As we expect of our reviewers, authors must avoid assuming intent with whom they disagree and never present ad hominem attacks. Assuming anything about those with whom you disagree is simply bullying and bigotry. Yes, we use strong language here because authors have the last word once something is in print. It is especially important for authors to maintain civil discourse given the best of science will often contradict or disagree with the work of others. Afterall, honest disagreement is the fuel of science.

In general, the papers we receive and the reviews that are provided are very respectful and appropriate. However, it feels timely to remind ourselves that we are a diverse community, but we are all experiencing uncertainty at a scale unlike most of the past. In stressful times such as these, civility and respect are important. Authors of papers may be more anxious and suspicious than was true in the past. The reviewer community is overworked and thus often rushed and naturally fallible. As editors, we wish to avoid censorship of reviews or papers. We respect the effort of all, and the good will of the academic community. Diversity is the cornerstone of our science, and we recognize that diversity extends to opinions, style, ideas, and practice. So, in times such as these having empathy for others is appreciated. We remain an author‐friendly journal, and we strive to be a reviewer‐, author‐, and user‐friendly journal overall. We pledge to you, as the Editor‐in‐Chief and Executive Editor, to strive to apply these standards in all that we do at *Ecology and Evolution*.

## Author Contributions

**Allen J. Moore:** conceptualization (lead), writing – original draft (lead), writing – review and editing (equal). **Arley Muth:** conceptualization (supporting), writing – original draft (supporting), writing – review and editing (equal).

## Conflicts of Interest

The authors declare no conflicts of interest.

## Data Availability

The authors have nothing to report.

